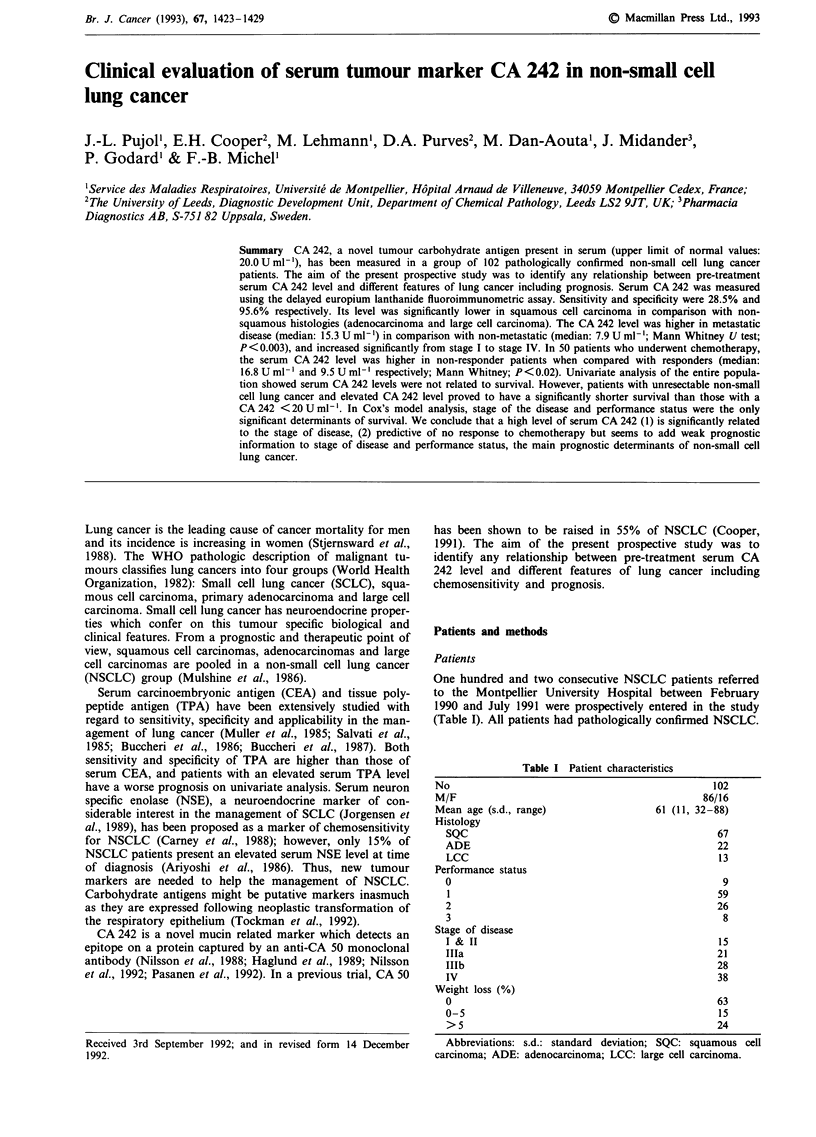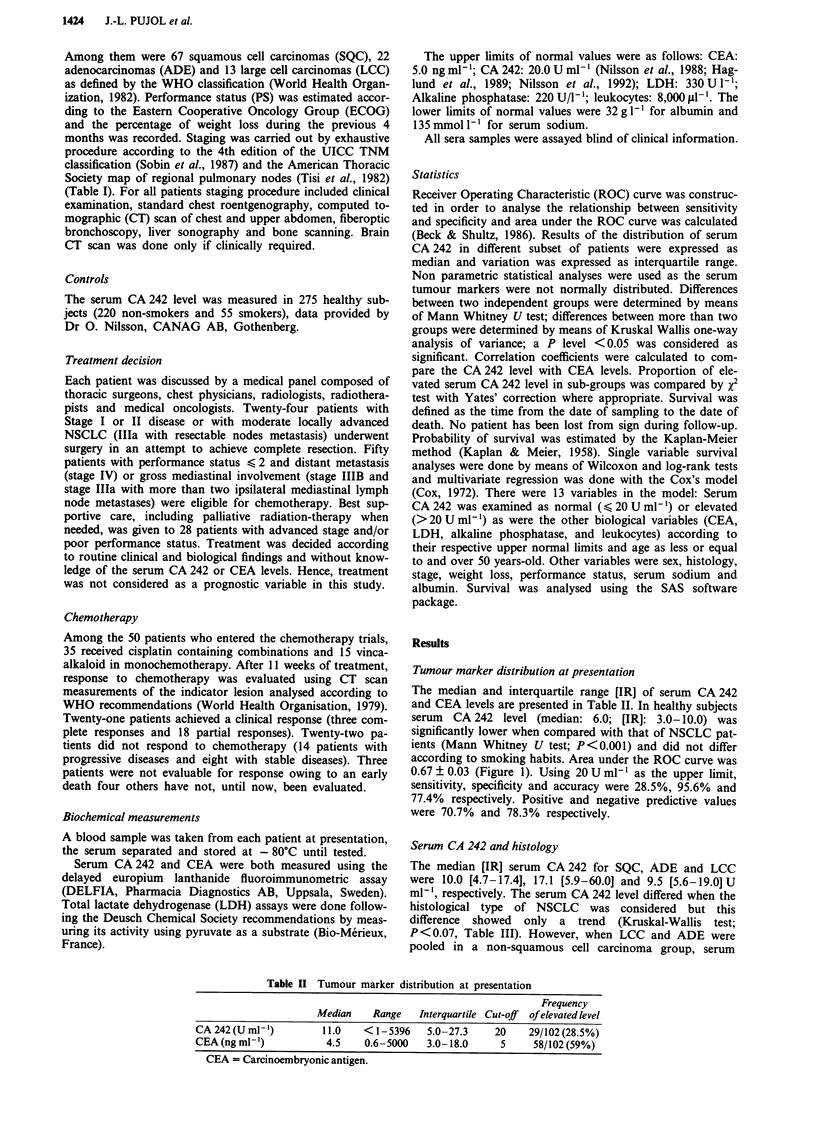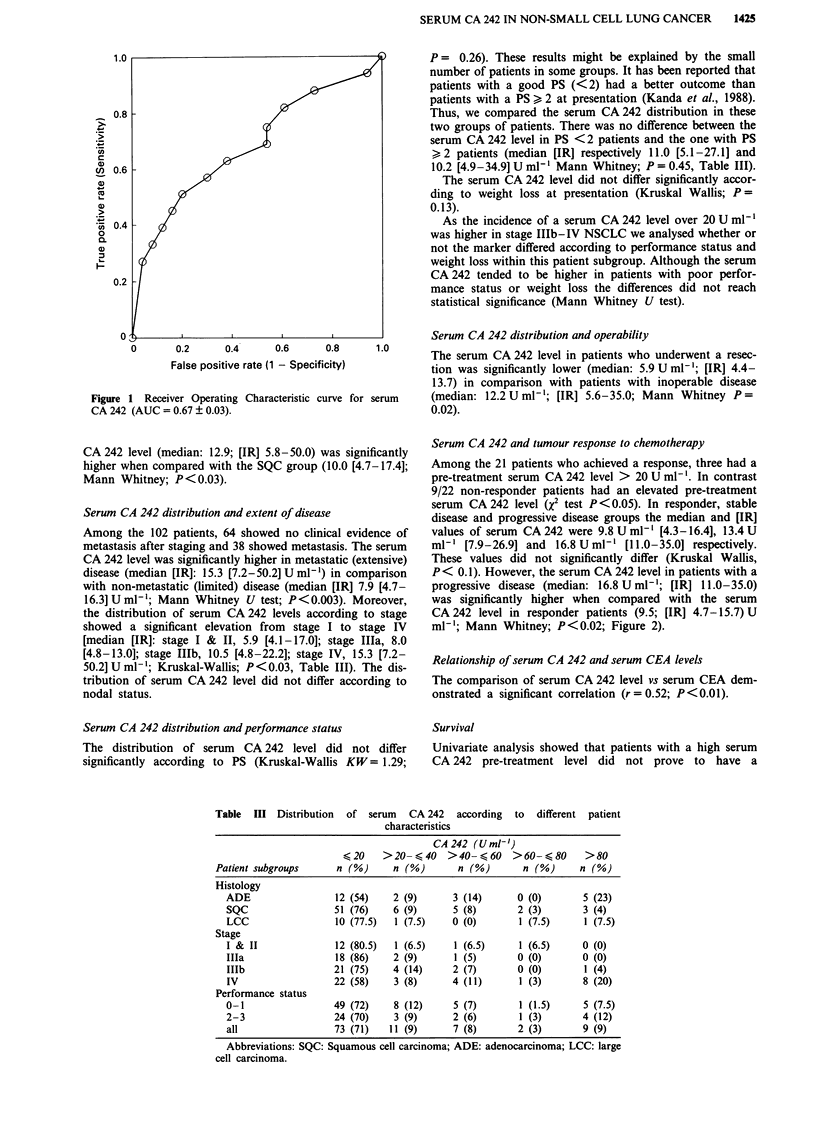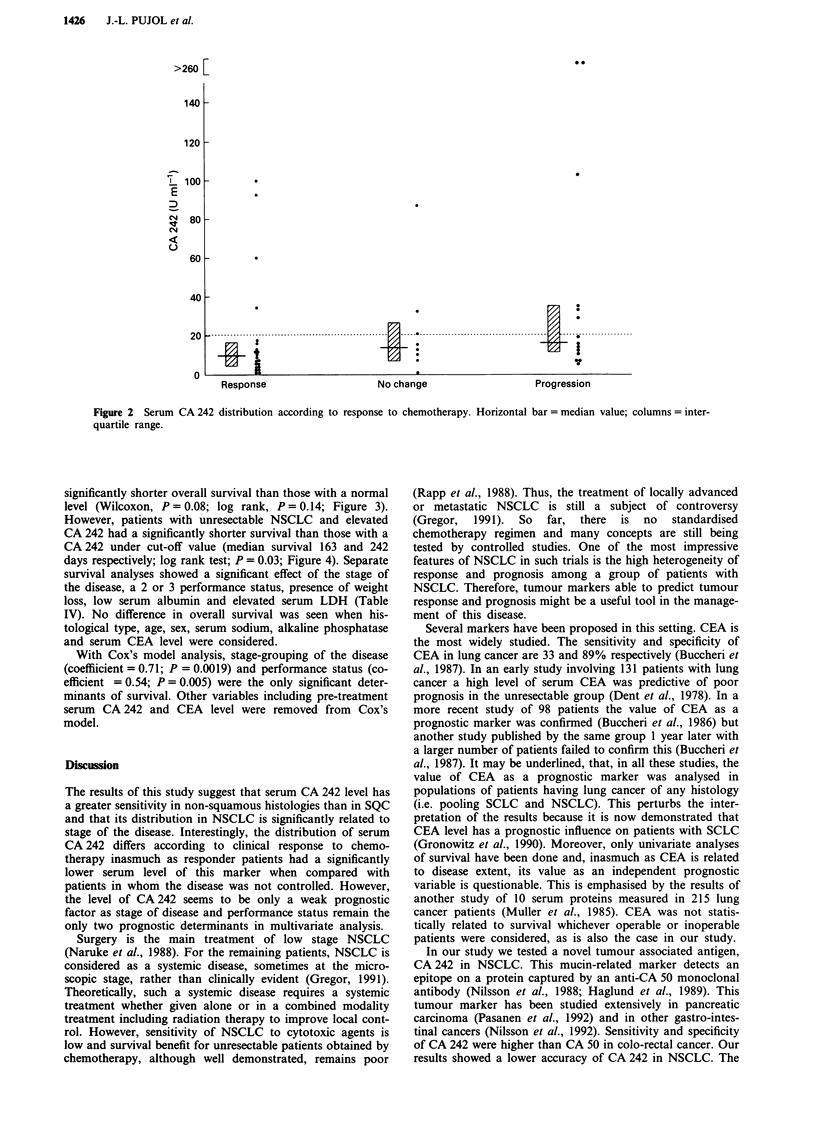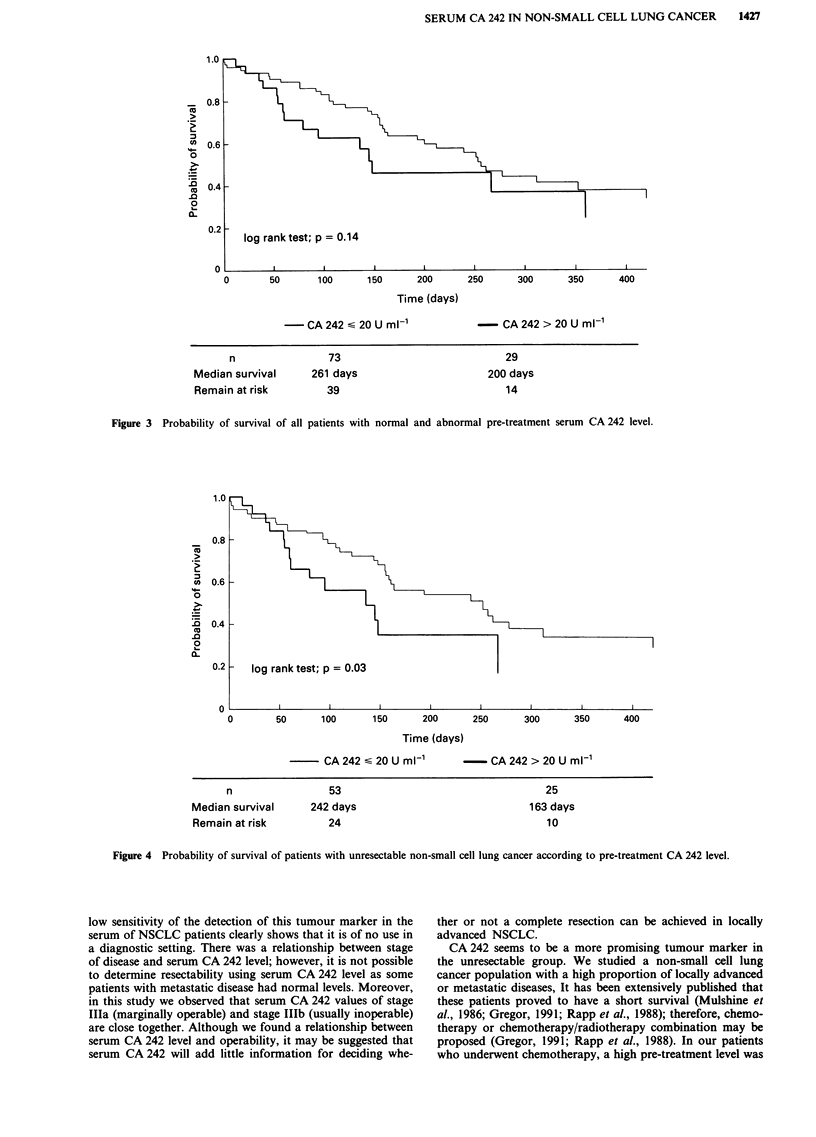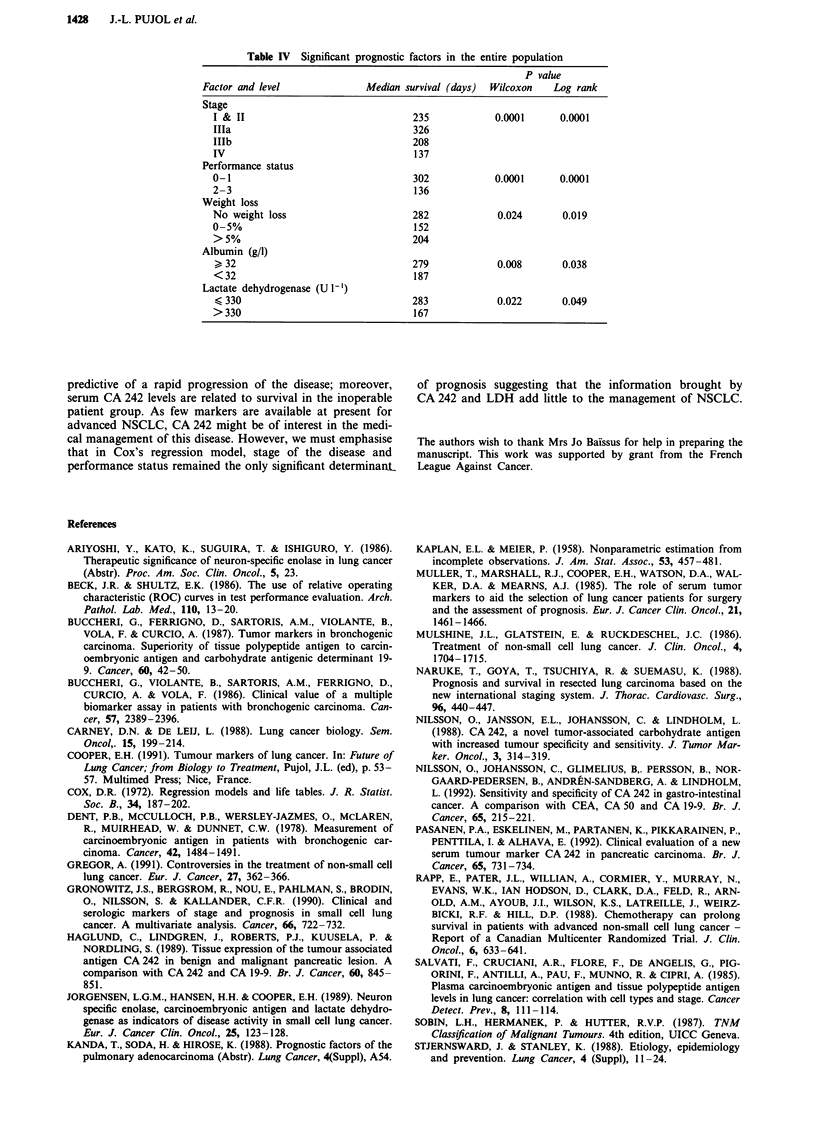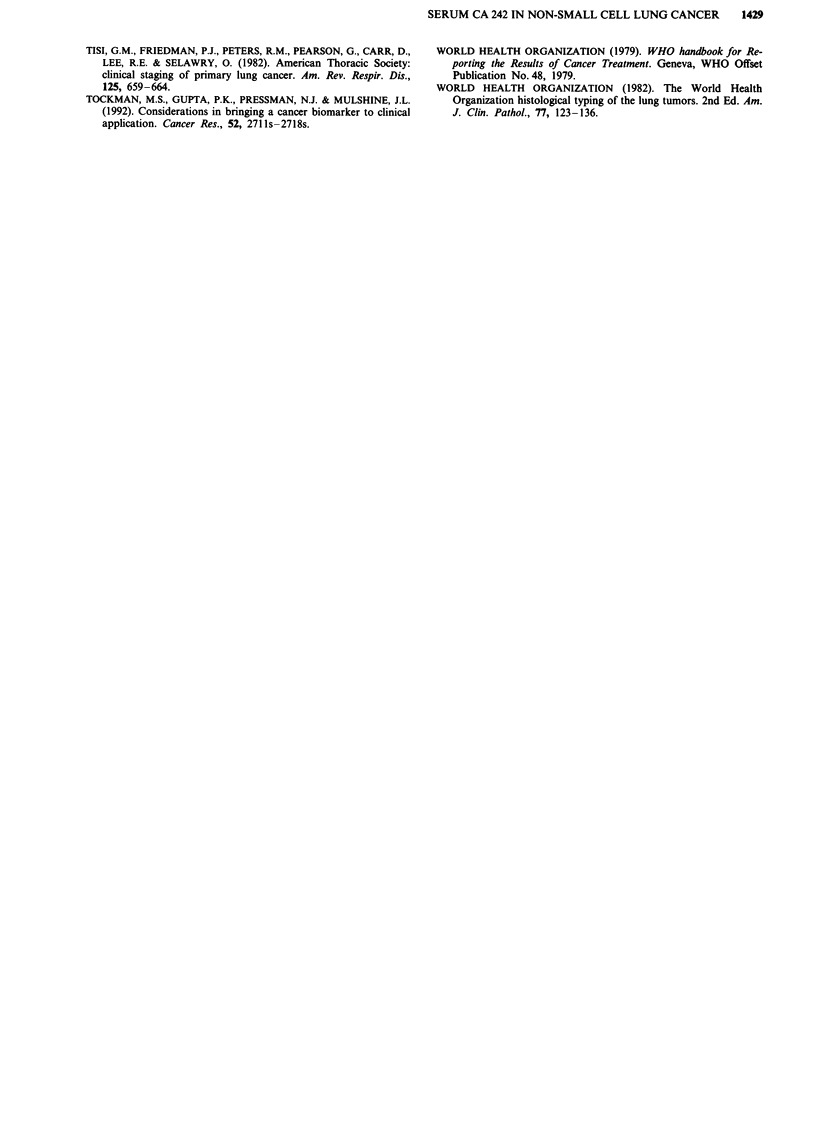# Clinical evaluation of serum tumour marker CA 242 in non-small cell lung cancer.

**DOI:** 10.1038/bjc.1993.264

**Published:** 1993-06

**Authors:** J. L. Pujol, E. H. Cooper, M. Lehmann, D. A. Purves, M. Dan-Aouta, J. Midander, P. Godard, F. B. Michel

**Affiliations:** Service des Maladies Respiratoires, Université de Montpellier, Hôpital Arnaud de Villeneuve, France.

## Abstract

CA 242, a novel tumour carbohydrate antigen present in serum (upper limit of normal values: 20.0 U ml-1), has been measured in a group of 102 pathologically confirmed non-small cell lung cancer patients. The aim of the present prospective study was to identify any relationship between pre-treatment serum CA 242 level and different features of lung cancer including prognosis. Serum CA 242 was measured using the delayed europium lanthanide fluoroimmunometric assay. Sensitivity and specificity were 28.5% and 95.6% respectively. Its level was significantly lower in squamous cell carcinoma in comparison with non-squamous histologies (adenocarcinoma and large cell carcinoma). The CA 242 level was higher in metastatic disease (median: 15.3 U ml-1) in comparison with non-metastatic (median: 7.9 U ml-1; Mann Whitney U test; P < 0.003), and increased significantly from stage I to stage IV. In 50 patients who underwent chemotherapy, the serum CA 242 level was higher in non-responder patients when compared with responders (median: 16.8 U ml-1 and 9.5 U ml-1 respectively; Mann Whitney; P < 0.02). Univariate analysis of the entire population showed serum CA 242 levels were not related to survival. However, patients with unresectable non-small cell lung cancer and elevated CA 242 level proved to have a significantly shorter survival than those with a CA 242 < 20 U ml-1. In Cox's model analysis, stage of the disease and performance status were the only significant determinants of survival. We conclude that a high level of serum CA 242 (1) is significantly related to the stage of disease, (2) predictive of no response to chemotherapy but seems to add weak prognostic information to stage of disease and performance status, the main prognostic determinants of non-small cell lung cancer.


Br. J. Cancer (1993), 67, 1423–1429 © Macmillan Press Ltd., 1993

### Clinical evaluation of serum tumour marker CA 242 in non-small cell lung cancer

J.-L. Pujol[1], E.H. Cooper[2], M. Lehmann[1], D.A. Purves[2], M. Dan-Aouta[1], J. Midander[3], P. Godard[1] & F.-B. Michel[1]

[1]*Service des Maladies Respiratoires, Université de Montpellier, Hôpital Arnaud de Villeneuve, 34059 Montpellier Cedex, France;* [2]*The University of Leeds, Diagnostic Development Unit, Department of Chemical Pathology, Leeds LS2 9JT, UK;* [3]*Pharmacia Diagnostics AB, S-751 82 Uppsala, Sweden.*

**Summary** CA 242, a novel tumour carbohydrate antigen present in serum (upper limit of normal values: 20.0 U $ml^{-1}$), has been measured in a group of 102 pathologically confirmed non-small cell lung cancer patients. The aim of the present prospective study was to identify any relationship between pre-treatment serum CA 242 level and different features of lung cancer including prognosis. Serum CA 242 was measured using the delayed europium lanthanide fluoroimmunometric assay. Sensitivity and specificity were 28.5% and 95.6% respectively. Its level was significantly lower in squamous cell carcinoma in comparison with non-squamous histologies (adenocarcinoma and large cell carcinoma). The CA 242 level was higher in metastatic disease (median: 15.3 U $ml^{-1}$) in comparison with non-metastatic (median: 7.9 U $ml^{-1}$; Mann Whitney $U$ test; $P<0.003$), and increased significantly from stage I to stage IV. In 50 patients who underwent chemotherapy, the serum CA 242 level was higher in non-responder patients when compared with responders (median: 16.8 U $ml^{-1}$ and 9.5 U $ml^{-1}$ respectively; Mann Whitney; $P<0.02$). Univariate analysis of the entire population showed serum CA 242 levels were not related to survival. However, patients with unresectable non-small cell lung cancer and elevated CA 242 level proved to have a significantly shorter survival than those with a CA 242 $<20$ U $ml^{-1}$. In Cox's model analysis, stage of the disease and performance status were the only significant determinants of survival. We conclude that a high level of serum CA 242 (1) is significantly related to the stage of disease, (2) predictive of no response to chemotherapy but seems to add weak prognostic information to stage of disease and performance status, the main prognostic determinants of non-small cell lung cancer.

Lung cancer is the leading cause of cancer mortality for men and its incidence is increasing in women (Stjernsward *et al.*, 1988). The WHO pathologic description of malignant tumours classifies lung cancers into four groups (World Health Organization, 1982): Small cell lung cancer (SCLC), squamous cell carcinoma, primary adenocarcinoma and large cell carcinoma. Small cell lung cancer has neuroendocrine properties which confer on this tumour specific biological and clinical features. From a prognostic and therapeutic point of view, squamous cell carcinomas, adenocarcinomas and large cell carcinomas are pooled in a non-small cell lung cancer (NSCLC) group (Mulshine *et al.*, 1986).

Serum carcinoembryonic antigen (CEA) and tissue polypeptide antigen (TPA) have been extensively studied with regard to sensitivity, specificity and applicability in the management of lung cancer (Muller *et al.*, 1985; Salvati *et al.*, 1985; Buccheri *et al.*, 1986; Buccheri *et al.*, 1987). Both sensitivity and specificity of TPA are higher than those of serum CEA, and patients with an elevated serum TPA level have a worse prognosis on univariate analysis. Serum neuron specific enolase (NSE), a neuroendocrine marker of considerable interest in the management of SCLC (Jorgensen *et al.*, 1989), has been proposed as a marker of chemosensitivity for NSCLC (Carney *et al.*, 1988); however, only 15% of NSCLC patients present an elevated serum NSE level at time of diagnosis (Ariyoshi *et al.*, 1986). Thus, new tumour markers are needed to help the management of NSCLC. Carbohydrate antigens might be putative markers inasmuch as they are expressed following neoplastic transformation of the respiratory epithelium (Tockman *et al.*, 1992).

CA 242 is a novel mucin related marker which detects an epitope on a protein captured by an anti-CA 50 monoclonal antibody (Nilsson *et al.*, 1988; Haglund *et al.*, 1989; Nilsson *et al.*, 1992; Pasanen *et al.*, 1992). In a previous trial, CA 50 has been shown to be raised in 55% of NSCLC (Cooper, 1991). The aim of the present prospective study was to identify any relationship between pre-treatment serum CA 242 level and different features of lung cancer including chemosensitivity and prognosis.

### Patients and methods

### *Patients*

One hundred and two consecutive NSCLC patients referred to the Montpellier University Hospital between February 1990 and July 1991 were prospectively entered in the study (Table I). All patients had pathologically confirmed NSCLC.

**Table I** Patient characteristics

| | |
|---|---|
| No | 102 |
| M/F | 86/16 |
| Mean age (s.d., range) | 61 (11, 32–88) |
| Histology | |
| SQC | 67 |
| ADE | 22 |
| LCC | 13 |
| Performance status | |
| 0 | 9 |
| 1 | 59 |
| 2 | 26 |
| 3 | 8 |
| Stage of disease | |
| I & II | 15 |
| IIIa | 21 |
| IIIb | 28 |
| IV | 38 |
| Weight loss (%) | |
| 0 | 63 |
| 0–5 | 15 |
| >5 | 24 |

Abbreviations: s.d.: standard deviation; SQC: squamous cell carcinoma; ADE: adenocarcinoma; LCC: large cell carcinoma.

Received 3rd September 1992; and in revised form 14 December 1992.

Among them were 67 squamous cell carcinomas (SQC), 22 adenocarcinomas (ADE) and 13 large cell carcinomas (LCC) as defined by the WHO classification (World Health Organization, 1982). Performance status (PS) was estimated according to the Eastern Cooperative Oncology Group (ECOG) and the percentage of weight loss during the previous 4 months was recorded. Staging was carried out by exhaustive procedure according to the 4th edition of the UICC TNM classification (Sobin et al., 1987) and the American Thoracic Society map of regional pulmonary nodes (Tisi et al., 1982) (Table I). For all patients staging procedure included clinical examination, standard chest roentgenography, computed tomographic (CT) scan of chest and upper abdomen, fiberoptic bronchoscopy, liver sonography and bone scanning. Brain CT scan was done only if clinically required.

*Controls*

The serum CA 242 level was measured in 275 healthy subjects (220 non-smokers and 55 smokers), data provided by Dr O. Nilsson, CANAG AB, Gothenberg.

*Treatment decision*

Each patient was discussed by a medical panel composed of thoracic surgeons, chest physicians, radiologists, radiotherapists and medical oncologists. Twenty-four patients with Stage I or II disease or with moderate locally advanced NSCLC (IIIa with resectable nodes metastasis) underwent surgery in an attempt to achieve complete resection. Fifty patients with performance status $\leqslant 2$ and distant metastasis (stage IV) or gross mediastinal involvement (stage IIIB and stage IIIa with more than two ipsilateral mediastinal lymph node metastases) were eligible for chemotherapy. Best supportive care, including palliative radiation-therapy when needed, was given to 28 patients with advanced stage and/or poor performance status. Treatment was decided according to routine clinical and biological findings and without knowledge of the serum CA 242 or CEA levels. Hence, treatment was not considered as a prognostic variable in this study.

*Chemotherapy*

Among the 50 patients who entered the chemotherapy trials, 35 received cisplatin containing combinations and 15 vinca-alkaloid in monochemotherapy. After 11 weeks of treatment, response to chemotherapy was evaluated using CT scan measurements of the indicator lesion analysed according to WHO recommendations (World Health Organisation, 1979). Twenty-one patients achieved a clinical response (three complete responses and 18 partial responses). Twenty-two patients did not respond to chemotherapy (14 patients with progressive diseases and eight with stable diseases). Three patients were not evaluable for response owing to an early death four others have not, until now, been evaluated.

*Biochemical measurements*

A blood sample was taken from each patient at presentation, the serum separated and stored at $-80°C$ until tested.

Serum CA 242 and CEA were both measured using the delayed europium lanthanide fluoroimmunometric assay (DELFIA, Pharmacia Diagnostics AB, Uppsala, Sweden). Total lactate dehydrogenase (LDH) assays were done following the Deusch Chemical Society recommendations by measuring its activity using pyruvate as a substrate (Bio-Mérieux, France).

The upper limits of normal values were as follows: CEA: $5.0\ ng\ ml^{-1}$; CA 242: $20.0\ U\ ml^{-1}$ (Nilsson et al., 1988; Haglund et al., 1989; Nilsson et al., 1992); LDH: $330\ U\ l^{-1}$; Alkaline phosphatase: $220\ U/l^{-1}$; leukocytes: $8,000\ \mu l^{-1}$. The lower limits of normal values were $32\ g\ l^{-1}$ for albumin and $135\ mmol\ l^{-1}$ for serum sodium.

All sera samples were assayed blind of clinical information.

*Statistics*

Receiver Operating Characteristic (ROC) curve was constructed in order to analyse the relationship between sensitivity and specificity and area under the ROC curve was calculated (Beck & Shultz, 1986). Results of the distribution of serum CA 242 in different subset of patients were expressed as median and variation was expressed as interquartile range. Non parametric statistical analyses were used as the serum tumour markers were not normally distributed. Differences between two independent groups were determined by means of Mann Whitney $U$ test; differences between more than two groups were determined by means of Kruskal Wallis one-way analysis of variance; a $P$ level $<0.05$ was considered as significant. Correlation coefficients were calculated to compare the CA 242 level with CEA levels. Proportion of elevated serum CA 242 level in sub-groups was compared by $\chi^2$ test with Yates' correction where appropriate. Survival was defined as the time from the date of sampling to the date of death. No patient has been lost from sign during follow-up. Probability of survival was estimated by the Kaplan-Meier method (Kaplan & Meier, 1958). Single variable survival analyses were done by means of Wilcoxon and log-rank tests and multivariate regression was done with the Cox's model (Cox, 1972). There were 13 variables in the model: Serum CA 242 was examined as normal ($\leqslant 20\ U\ ml^{-1}$) or elevated ($>20\ U\ ml^{-1}$) as were the other biological variables (CEA, LDH, alkaline phosphatase, and leukocytes) according to their respective upper normal limits and age as less or equal to and over 50 years-old. Other variables were sex, histology, stage, weight loss, performance status, serum sodium and albumin. Survival was analysed using the SAS software package.

### Results

*Tumour marker distribution at presentation*

The median and interquartile range [IR] of serum CA 242 and CEA levels are presented in Table II. In healthy subjects serum CA 242 level (median: 6.0; [IR]: 3.0–10.0) was significantly lower when compared with that of NSCLC patients (Mann Whitney $U$ test; $P<0.001$) and did not differ according to smoking habits. Area under the ROC curve was $0.67 \pm 0.03$ (Figure 1). Using $20\ U\ ml^{-1}$ as the upper limit, sensitivity, specificity and accuracy were 28.5%, 95.6% and 77.4% respectively. Positive and negative predictive values were 70.7% and 78.3% respectively.

*Serum CA 242 and histology*

The median [IR] serum CA 242 for SQC, ADE and LCC were 10.0 [4.7–17.4], 17.1 [5.9–60.0] and 9.5 [5.6–19.0] $U\ ml^{-1}$, respectively. The serum CA 242 level differed when the histological type of NSCLC was considered but this difference showed only a trend (Kruskal-Wallis test; $P<0.07$, Table III). However, when LCC and ADE were pooled in a non-squamous cell carcinoma group, serum

**Table II** Tumour marker distribution at presentation

| | *Median* | *Range* | *Interquartile* | *Cut-off* | *Frequency of elevated level* |
|---|---|---|---|---|---|
| CA 242 ($U\ ml^{-1}$) | 11.0 | <1–5396 | 5.0–27.3 | 20 | 29/102 (28.5%) |
| CEA ($ng\ ml^{-1}$) | 4.5 | 0.6–5000 | 3.0–18.0 | 5 | 58/102 (59%) |

CEA = Carcinoembryonic antigen.

**Figure 1** Receiver Operating Characteristic curve for serum CA 242 (AUC = 0.67 ± 0.03).

CA 242 level (median: 12.9; [IR] 5.8–50.0) was significantly higher when compared with the SQC group (10.0 [4.7–17.4]; Mann Whitney; $P<0.03$).

*Serum CA 242 distribution and extent of disease*

Among the 102 patients, 64 showed no clinical evidence of metastasis after staging and 38 showed metastasis. The serum CA 242 level was significantly higher in metastatic (extensive) disease (median [IR]: 15.3 [7.2–50.2] U $ml^{-1}$) in comparison with non-metastatic (limited) disease (median [IR] 7.9 [4.7–16.3] U $ml^{-1}$; Mann Whitney *U* test; $P<0.003$). Moreover, the distribution of serum CA 242 levels according to stage showed a significant elevation from stage I to stage IV [median [IR]: stage I & II, 5.9 [4.1–17.0]; stage IIIa, 8.0 [4.8–13.0]; stage IIIb, 10.5 [4.8–22.2]; stage IV, 15.3 [7.2–50.2] U $ml^{-1}$; Kruskal-Wallis; $P<0.03$, Table III). The distribution of serum CA 242 level did not differ according to nodal status.

*Serum CA 242 distribution and performance status*

The distribution of serum CA 242 level did not differ significantly according to PS (Kruskal-Wallis $KW=1.29$; $P=0.26$). These results might be explained by the small number of patients in some groups. It has been reported that patients with a good PS (<2) had a better outcome than patients with a PS ≥ 2 at presentation (Kanda *et al.*, 1988). Thus, we compared the serum CA 242 distribution in these two groups of patients. There was no difference between the serum CA 242 level in PS <2 patients and the one with PS ≥ 2 patients (median [IR] respectively 11.0 [5.1–27.1] and 10.2 [4.9–34.9] U $ml^{-1}$ Mann Whitney; $P=0.45$, Table III).

The serum CA 242 level did not differ significantly according to weight loss at presentation (Kruskal Wallis; $P=0.13$).

As the incidence of a serum CA 242 level over 20 U $ml^{-1}$ was higher in stage IIIb–IV NSCLC we analysed whether or not the marker differed according to performance status and weight loss within this patient subgroup. Although the serum CA 242 tended to be higher in patients with poor performance status or weight loss the differences did not reach statistical significance (Mann Whitney *U* test).

*Serum CA 242 distribution and operability*

The serum CA 242 level in patients who underwent a resection was significantly lower (median: 5.9 U $ml^{-1}$; [IR] 4.4–13.7) in comparison with patients with inoperable disease (median: 12.2 U $ml^{-1}$; [IR] 5.6–35.0; Mann Whitney $P=0.02$).

*Serum CA 242 and tumour response to chemotherapy*

Among the 21 patients who achieved a response, three had a pre-treatment serum CA 242 level > 20 U $ml^{-1}$. In contrast 9/22 non-responder patients had an elevated pre-treatment serum CA 242 level ($\chi^2$ test $P<0.05$). In responder, stable disease and progressive disease groups the median and [IR] values of serum CA 242 were 9.8 U $ml^{-1}$ [4.3–16.4], 13.4 U $ml^{-1}$ [7.9–26.9] and 16.8 U $ml^{-1}$ [11.0–35.0] respectively. These values did not significantly differ (Kruskal Wallis, $P<0.1$). However, the serum CA 242 level in patients with a progressive disease (median: 16.8 U $ml^{-1}$; [IR] 11.0–35.0) was significantly higher when compared with the serum CA 242 level in responder patients (9.5; [IR] 4.7–15.7) U $ml^{-1}$; Mann Whitney; $P<0.02$; Figure 2).

*Relationship of serum CA 242 and serum CEA levels*

The comparison of serum CA 242 level *vs* serum CEA demonstrated a significant correlation ($r=0.52$; $P<0.01$).

*Survival*

Univariate analysis showed that patients with a high serum CA 242 pre-treatment level did not prove to have a

**Table III** Distribution of serum CA 242 according to different patient characteristics

| | *CA 242 (U ml⁻¹)* | | | | |
|---|---|---|---|---|---|
| | *≤20* | *>20–≤40* | *>40–≤60* | *>60–≤80* | *>80* |
| *Patient subgroups* | *n (%)* | *n (%)* | *n (%)* | *n (%)* | *n (%)* |
| Histology | | | | | |
| ADE | 12 (54) | 2 (9) | 3 (14) | 0 (0) | 5 (23) |
| SQC | 51 (76) | 6 (9) | 5 (8) | 2 (3) | 3 (4) |
| LCC | 10 (77.5) | 1 (7.5) | 0 (0) | 1 (7.5) | 1 (7.5) |
| Stage | | | | | |
| I & II | 12 (80.5) | 1 (6.5) | 1 (6.5) | 1 (6.5) | 0 (0) |
| IIIa | 18 (86) | 2 (9) | 1 (5) | 0 (0) | 0 (0) |
| IIIb | 21 (75) | 4 (14) | 2 (7) | 0 (0) | 1 (4) |
| IV | 22 (58) | 3 (8) | 4 (11) | 1 (3) | 8 (20) |
| Performance status | | | | | |
| 0–1 | 49 (72) | 8 (12) | 5 (7) | 1 (1.5) | 5 (7.5) |
| 2–3 | 24 (70) | 3 (9) | 2 (6) | 1 (3) | 4 (12) |
| all | 73 (71) | 11 (9) | 7 (8) | 2 (3) | 9 (9) |

Abbreviations: SQC: Squamous cell carcinoma; ADE: adenocarcinoma; LCC: large cell carcinoma.

**Figure 2** Serum CA 242 distribution according to response to chemotherapy. Horizontal bar = median value; columns = interquartile range.

significantly shorter overall survival than those with a normal level (Wilcoxon, $P = 0.08$; log rank, $P = 0.14$; Figure 3). However, patients with unresectable NSCLC and elevated CA 242 had a significantly shorter survival than those with a CA 242 under cut-off value (median survival 163 and 242 days respectively; log rank test; $P = 0.03$; Figure 4). Separate survival analyses showed a significant effect of the stage of the disease, a 2 or 3 performance status, presence of weight loss, low serum albumin and elevated serum LDH (Table IV). No difference in overall survival was seen when histological type, age, sex, serum sodium, alkaline phosphatase and serum CEA level were considered.

With Cox's model analysis, stage-grouping of the disease (coeffiicient = 0.71; $P = 0.0019$) and performance status (coefficient = 0.54; $P = 0.005$) were the only significant determinants of survival. Other variables including pre-treatment serum CA 242 and CEA level were removed from Cox's model.

### Discussion

The results of this study suggest that serum CA 242 level has a greater sensitivity in non-squamous histologies than in SQC and that its distribution in NSCLC is significantly related to stage of the disease. Interestingly, the distribution of serum CA 242 differs according to clinical response to chemotherapy inasmuch as responder patients had a significantly lower serum level of this marker when compared with patients in whom the disease was not controlled. However, the level of CA 242 seems to be only a weak prognostic factor as stage of disease and performance status remain the only two prognostic determinants in multivariate analysis.

Surgery is the main treatment of low stage NSCLC (Naruke et al., 1988). For the remaining patients, NSCLC is considered as a systemic disease, sometimes at the microscopic stage, rather than clinically evident (Gregor, 1991). Theoretically, such a systemic disease requires a systemic treatment whether given alone or in a combined modality treatment including radiation therapy to improve local control. However, sensitivity of NSCLC to cytotoxic agents is low and survival benefit for unresectable patients obtained by chemotherapy, although well demonstrated, remains poor (Rapp et al., 1988). Thus, the treatment of locally advanced or metastatic NSCLC is still a subject of controversy (Gregor, 1991). So far, there is no standardised chemotherapy regimen and many concepts are still being tested by controlled studies. One of the most impressive features of NSCLC in such trials is the high heterogeneity of response and prognosis among a group of patients with NSCLC. Therefore, tumour markers able to predict tumour response and prognosis might be a useful tool in the management of this disease.

Several markers have been proposed in this setting. CEA is the most widely studied. The sensitivity and specificity of CEA in lung cancer are 33 and 89% respectively (Buccheri et al., 1987). In an early study involving 131 patients with lung cancer a high level of serum CEA was predictive of poor prognosis in the unresectable group (Dent et al., 1978). In a more recent study of 98 patients the value of CEA as a prognostic marker was confirmed (Buccheri et al., 1986) but another study published by the same group 1 year later with a larger number of patients failed to confirm this (Buccheri et al., 1987). It may be underlined, that, in all these studies, the value of CEA as a prognostic marker was analysed in populations of patients having lung cancer of any histology (i.e. pooling SCLC and NSCLC). This perturbs the interpretation of the results because it is now demonstrated that CEA level has a prognostic influence on patients with SCLC (Gronowitz et al., 1990). Moreover, only univariate analyses of survival have been done and, inasmuch as CEA is related to disease extent, its value as an independent prognostic variable is questionable. This is emphasised by the results of another study of 10 serum proteins measured in 215 lung cancer patients (Muller et al., 1985). CEA was not statistically related to survival whichever operable or inoperable patients were considered, as is also the case in our study.

In our study we tested a novel tumour associated antigen, CA 242 in NSCLC. This mucin-related marker detects an epitope on a protein captured by an anti-CA 50 monoclonal antibody (Nilsson et al., 1988; Haglund et al., 1989). This tumour marker has been studied extensively in pancreatic carcinoma (Pasanen et al., 1992) and in other gastro-intestinal cancers (Nilsson et al., 1992). Sensitivity and specificity of CA 242 were higher than CA 50 in colo-rectal cancer. Our results showed a lower accuracy of CA 242 in NSCLC. The

| | | |
|---|---|---|
| n | 73 | 29 |
| Median survival | 261 days | 200 days |
| Remain at risk | 39 | 14 |

**Figure 3** Probability of survival of all patients with normal and abnormal pre-treatment serum CA 242 level.

| | | |
|---|---|---|
| n | 53 | 25 |
| Median survival | 242 days | 163 days |
| Remain at risk | 24 | 10 |

**Figure 4** Probability of survival of patients with unresectable non-small cell lung cancer according to pre-treatment CA 242 level.

low sensitivity of the detection of this tumour marker in the serum of NSCLC patients clearly shows that it is of no use in a diagnostic setting. There was a relationship between stage of disease and serum CA 242 level; however, it is not possible to determine resectability using serum CA 242 level as some patients with metastatic disease had normal levels. Moreover, in this study we observed that serum CA 242 values of stage IIIa (marginally operable) and stage IIIb (usually inoperable) are close together. Although we found a relationship between serum CA 242 level and operability, it may be suggested that serum CA 242 will add little information for deciding whether or not a complete resection can be achieved in locally advanced NSCLC.

CA 242 seems to be a more promising tumour marker in the unresectable group. We studied a non-small cell lung cancer population with a high proportion of locally advanced or metastatic diseases, It has been extensively published that these patients proved to have a short survival (Mulshine *et al.*, 1986; Gregor, 1991; Rapp *et al.*, 1988); therefore, chemotherapy or chemotherapy/radiotherapy combination may be proposed (Gregor, 1991; Rapp *et al.*, 1988). In our patients who underwent chemotherapy, a high pre-treatment level was

**Table IV** Significant prognostic factors in the entire population

| Factor and level | Median survival (days) | P value Wilcoxon | Log rank |
|---|---|---|---|
| Stage | | | |
| I & II | 235 | 0.0001 | 0.0001 |
| IIIa | 326 | | |
| IIIb | 208 | | |
| IV | 137 | | |
| Performance status | | | |
| 0–1 | 302 | 0.0001 | 0.0001 |
| 2–3 | 136 | | |
| Weight loss | | | |
| No weight loss | 282 | 0.024 | 0.019 |
| 0–5% | 152 | | |
| >5% | 204 | | |
| Albumin (g/l) | | | |
| ⩾32 | 279 | 0.008 | 0.038 |
| <32 | 187 | | |
| Lactate dehydrogenase ($U\,l^{-1}$) | | | |
| ⩽330 | 283 | 0.022 | 0.049 |
| >330 | 167 | | |

predictive of a rapid progression of the disease; moreover, serum CA 242 levels are related to survival in the inoperable patient group. As few markers are available at present for advanced NSCLC, CA 242 might be of interest in the medical management of this disease. However, we must emphasise that in Cox's regression model, stage of the disease and performance status remained the only significant determinant of prognosis suggesting that the information brought by CA 242 and LDH add little to the management of NSCLC.

The authors wish to thank Mrs Jo Baïssus for help in preparing the manuscript. This work was supported by grant from the French League Against Cancer.

### References

ARIYOSHI, Y., KATO, K., SUGUIRA, T. & ISHIGURO, Y. (1986). Therapeutic significance of neuron-specific enolase in lung cancer (Abstr). *Proc. Am. Soc. Clin. Oncol.*, **5**, 23.

BECK, J.R. & SHULTZ, E.K. (1986). The use of relative operating characteristic (ROC) curves in test performance evaluation. *Arch. Pathol. Lab. Med.*, **110**, 13–20.

BUCCHERI, G., FERRIGNO, D., SARTORIS, A.M., VIOLANTE, B., VOLA, F. & CURCIO, A. (1987). Tumor markers in bronchogenic carcinoma. Superiority of tissue polypeptide antigen to carcinoembryonic antigen and carbohydrate antigenic determinant 19-9. *Cancer*, **60**, 42–50.

BUCCHERI, G., VIOLANTE, B., SARTORIS, A.M., FERRIGNO, D., CURCIO, A. & VOLA, F. (1986). Clinical value of a multiple biomarker assay in patients with bronchogenic carcinoma. *Cancer*, **57**, 2389–2396.

CARNEY, D.N. & DE LEIJ, L. (1988). Lung cancer biology. *Sem. Oncol*,. **15**, 199–214.

COOPER, E.H. (1991). Tumour markers of lung cancer. In: *Future of Lung Cancer; from Biology to Treatment*, Pujol, J.L. (ed), p. 53–57. Multimed Press; Nice, France.

COX, D.R. (1972). Regression models and life tables. *J. R. Statist. Soc. B.*, **34**, 187–202.

DENT, P.B., MCCULLOCH, P.B., WERSLEY-JAZMES, O., MCLAREN, R., MUIRHEAD, W. & DUNNET, C.W. (1978). Measurement of carcinoembryonic antigen in patients with bronchogenic carcinoma. *Cancer*, **42**, 1484–1491.

GREGOR, A. (1991). Controversies in the treatment of non-small cell lung cancer. *Eur. J. Cancer*, **27**, 362–366.

GRONOWITZ, J.S., BERGSROM, R., NOU, E., PAHLMAN, S., BRODIN, O., NILSSON, S. & KALLANDER, C.F.R. (1990). Clinical and serologic markers of stage and prognosis in small cell lung cancer. A multivariate analysis. *Cancer*, **66**, 722–732.

HAGLUND, C., LINDGREN, J., ROBERTS, P.J., KUUSELA, P. & NORDLING, S. (1989). Tissue expression of the tumour associated antigen CA 242 in benign and malignant pancreatic lesion. A comparison with CA 242 and CA 19-9. *Br. J. Cancer*, **60**, 845–851.

JORGENSEN, L.G.M., HANSEN, H.H. & COOPER, E.H. (1989). Neuron specific enolase, carcinoembryonic antigen and lactate dehydrogenase as indicators of disease activity in small cell lung cancer. *Eur. J. Cancer Clin. Oncol.*, **25**, 123–128.

KANDA, T., SODA, H. & HIROSE, K. (1988). Prognostic factors of the pulmonary adenocarcinoma (Abstr). *Lung Cancer*, **4**(Suppl), A54.

KAPLAN, E.L. & MEIER, P. (1958). Nonparametric estimation from incomplete observations. *J. Am. Stat. Assoc.*, **53**, 457–481.

MULLER, T., MARSHALL, R.J., COOPER, E.H., WATSON, D.A., WALKER, D.A. & MEARNS, A.J. (1985). The role of serum tumor markers to aid the selection of lung cancer patients for surgery and the assessment of prognosis. *Eur. J. Cancer Clin. Oncol.*, **21**, 1461–1466.

MULSHINE, J.L., GLATSTEIN, E. & RUCKDESCHEL, J.C. (1986). Treatment of non-small cell lung cancer. *J. Clin. Oncol.*, **4**, 1704–1715.

NARUKE, T., GOYA, T., TSUCHIYA, R. & SUEMASU, K. (1988). Prognosis and survival in resected lung carcinoma based on the new international staging system. *J. Thorac. Cardiovasc. Surg.*, **96**, 440–447.

NILSSON, O., JANSSON, E.L., JOHANSSON, C. & LINDHOLM, L. (1988). CA 242, a novel tumor-associated carbohydrate antigen with increased tumour specificity and sensitivity. *J. Tumor Marker. Oncol.*, **3**, 314–319.

NILSSON, O., JOHANSSON, C., GLIMELIUS, B,. PERSSON, B., NORGAARD-PEDERSEN, B., ANDRÉN-SANDBERG, A. & LINDHOLM, L. (1992). Sensitivity and specificity of CA 242 in gastro-intestinal cancer. A comparison with CEA, CA 50 and CA 19-9. *Br. J. Cancer*, **65**, 215–221.

PASANEN, P.A., ESKELINEN, M., PARTANEN, K., PIKKARAINEN, P., PENTTILA, I. & ALHAVA, E. (1992). Clinical evaluation of a new serum tumour marker CA 242 in pancreatic carcinoma. *Br. J. Cancer*, **65**, 731–734.

RAPP, E., PATER, J.L., WILLIAN, A., CORMIER, Y., MURRAY, N., EVANS, W.K., IAN HODSON, D., CLARK, D.A., FELD, R., ARNOLD, A.M., AYOUB, J.I., WILSON, K.S., LATREILLE, J., WEIRZBICKI, R.F. & HILL, D.P. (1988). Chemotherapy can prolong survival in patients with advanced non-small cell lung cancer – Report of a Canadian Multicenter Randomized Trial. *J. Clin. Oncol.*, **6**, 633–641.

SALVATI, F., CRUCIANI, A.R., FLORE, F., DE ANGELIS, G., PIGORINI, F., ANTILLI, A., PAU, F., MUNNO, R. & CIPRI, A. (1985). Plasma carcinoembryonic antigen and tissue polypeptide antigen levels in lung cancer: correlation with cell types and stage. *Cancer Detect. Prev.*, **8**, 111–114.

SOBIN, L.H., HERMANEK, P. & HUTTER, R.V.P. (1987). *TNM Classification of Malignant Tumours*. 4th edition, UICC Geneva.

STJERNSWARD, J. & STANLEY, K. (1988). Etiology, epidemiology and prevention. *Lung Cancer*, **4** (Suppl), 11–24.

TISI, G.M., FRIEDMAN, P.J., PETERS, R.M., PEARSON, G., CARR, D., LEE, R.E. & SELAWRY, O. (1982). American Thoracic Society: clinical staging of primary lung cancer. *Am. Rev. Respir. Dis.*, **125,** 659–664.

TOCKMAN, M.S., GUPTA, P.K., PRESSMAN, N.J. & MULSHINE, J.L. (1992). Considerations in bringing a cancer biomarker to clinical application. *Cancer Res.*, **52,** 2711s–2718s.

WORLD HEALTH ORGANIZATION (1979). *WHO handbook for Reporting the Results of Cancer Treatment*. Geneva, WHO Offset Publication No. 48, 1979.

WORLD HEALTH ORGANIZATION (1982). The World Health Organization histological typing of the lung tumors. 2nd Ed. *Am. J. Clin. Pathol.*, **77,** 123–136.